# Transformation of intestinal stem cells into gastric stem cells on loss of transcription factor Cdx2

**DOI:** 10.1038/ncomms6728

**Published:** 2014-12-11

**Authors:** Salvatore Simmini, Monika Bialecka, Meritxell Huch, Lennart Kester, Marc van de Wetering, Toshiro Sato, Felix Beck, Alexander van Oudenaarden, Hans Clevers, Jacqueline Deschamps

**Affiliations:** 1 Hubrecht Institute and UMC Utrecht, Uppsalalaan 8 3584 CT Utrecht, the Netherlands; 2 University of Leicester, Department of Biochemistry, Leicester LE1 7RH, UK

## Abstract

The endodermal lining of the adult gastro-intestinal tract harbours stem cells that are responsible for the day-to-day regeneration of the epithelium. Stem cells residing in the pyloric glands of the stomach and in the small intestinal crypts differ in their differentiation programme and in the gene repertoire that they express. Both types of stem cells have been shown to grow from single cells into 3D structures (organoids) *in vitro*. We show that single adult Lgr5-positive stem cells, isolated from small intestinal organoids, require Cdx2 to maintain their intestinal identity and are converted cell-autonomously into pyloric stem cells in the absence of this transcription factor. Clonal descendants of *Cdx2*^*null*^ small intestinal stem cells enter the gastric differentiation program instead of producing intestinal derivatives. We show that the intestinal genetic programme is critically dependent on the single transcription factor encoding gene *Cdx2*.

## Abstract (web summary)

The adult gastro-intestinal tract harbours stem cells that differ in their differentiation programme and in the gene repertoire that they express. Here the authors show that single adult Lgr5-positive stem cells require Cdx2 to maintain their intestinal identity and are converted into pyloric stem cells in the absence of this transcription factor.

## Graphical abstract

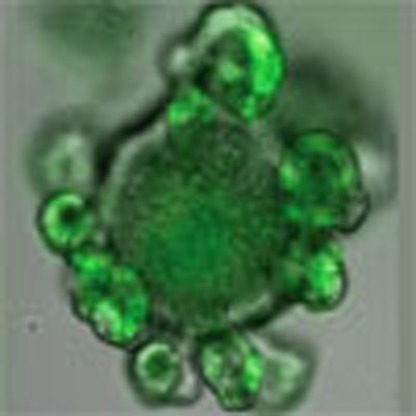

Adult stem cells from the digestive tract have been characterized *in vivo* and in organoid structures growing *in vitro*^1^,^2^,^3^. Although small intestinal stem cells and gastric stem cells both express the stem cell marker Lgr5, they each express their own set of stem cell markers^3^,^4^,^5^. In addition, stem cells of the gastric and small intestinal segments of the digestive tract already express some markers of their respective differentiation programme. Small intestinal stem cells (SI SCs) express low levels of Villin, Mucin2 and Lysozyme^5^, whereas gastric stem cells (Sto SCs) fail to express these markers and express low levels of gastric intrinsic factor (Gif)^3^. Therefore, they are already engaged in their tissue-specific differentiation programme. A comparison between the transcriptome of stem cells isolated from organs *in vivo* and from cultured organoids *in vitro* revealed the robustness and stability of these cells: each of these stem cells maintains its *in vivo* properties when cultured into organoids *in vitro*^2^,^3^.

We find that the properties and transcriptional signature of adult SI SCs cultured *in vitro* as organoids are critically dependent on the expression of the transcription factor Cdx2. We show that single SI SCs wherein Cdx2 was inactivated rapidly lose their intestinal identity and acquire a gastric pyloric identity. They cannot give rise to intestinal organoids *in vitro* as their wild-type counterparts do, and instead manifest growth properties and transcriptional profile of gastric pyloric SCs. *Cdx2*^*null*^ SI SCs exclusively express the transcriptional programme of gastric pyloric stem cells and generate differentiated derivatives of all pyloric lineages. These data indicate that Cdx2 is a major determinant of the identity and fate of adult small intestinal stem cells.

## Results

### Single *Cdx2*^*null*^ intestinal SCs form stomach organoids

It had been found that inactivation of the intestinal-specific transcription factor Cdx2 in the adult mouse intestinal epithelium leads to the transformation of some of the crypts into submucosal empty cysts expressing stomach markers^6^,^7^. This raised the fundamental question of whether the sole transcription factor Cdx2 was able to change the identity of adult intestinal stem cells into stem cells with a different commitment.

**Figure 1:**
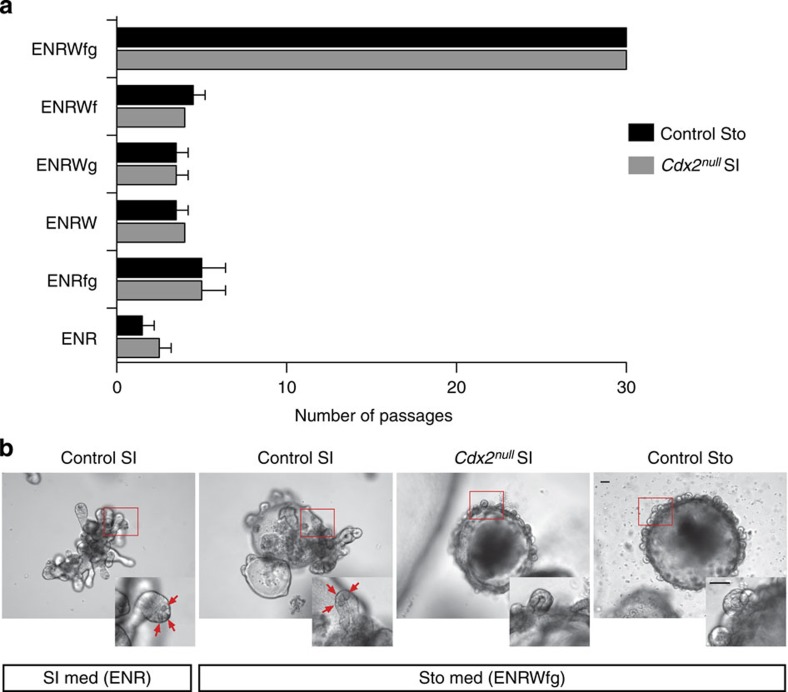
Isolated *Cdx2*^*null*^ SI SCs form gastric organoids. (**a**) Graph summarizing the growth performance (two independent experiments) of *Cdx2*^*null*^ SI SC-derived organoids and control Sto SC-derived organoids (issued from single Sto SCs) in medium dedicated to SI organoids (ENR, last rows of the graph, containing Egf, Noggin and R-Spondin1), in medium dedicated to Sto organoids (ENRWfg, top rows of the graphs, containing in addition to ENR, Wnt3a conditioned medium (W), Fgf10 (f) and Gastrin (g)), in SI medium supplemented with Fgf and Gastrin (ENRfg), SI medium supplemented with Wnt (ENRW), SI medium supplemented with Wnt and Gastrin (ENRWg) and SI medium supplemented with Wnt and Fgf (ENRWf). Black bars, stomach control organoids; dark grey bars, *Cdx2*^*null*^ SI organoids. *x*-axis, number of passages (one passage in average every 7 or 8 days). Error bars, s.d. (**b**) Control SI organoids in intestinal and stomach conditions (left two panels); insets, higher magnification of buds; red arrows, Paneth cells; *Cdx2*^*null*^ SI organoids (third panel from left) and control Sto organoids (right panel) in stomach conditions. Bars, 150 μm. med, medium. Images are representative of the results of more than 30 experiments.

We set out to investigate whether the ablation of *Cdx2* in Lgr5-positive stem cells isolated from adult small intestinal organoids would convert them into gastric stem cells. We used a stem cell (SC)-specific *Lgr5-EGFP-Ires-CreERT2* knock-in allele^1^ to inactivate *Cdx2* specifically in the stem cells of intestinal crypts cells. We induced inactivation of the floxed *Cdx2* allele^6^ in primary cultures of proximal small intestinal organoids derived from *Cdx2*^*−/fl*^*/Lgr5-EGFP-Ires-CreERT2* mice by overnight exposure to 4-hydroxytamoxifen^2^,^8^. After dissociation of the organoids, the Lgr5-EGFP^hi^ SI SCs were FACS-sorted, genotyped (Supplementary Fig. 1) and grown as single stem cell-derived clonal organoids. Unlike SI SCs from 4-hydroxytamoxifen-untreated *Cdx2*^*−/fl*^*/Lgr5-EGFP-Ires-CreERT2* organoids (from here on called control SI SCs), *Cdx2*^*null*^*/Lgr5-EGFP-Ires-CreERT2* SI SCs (from here on called *Cdx2*^*null*^ SI SCs) did not grow and form organoids in conditions established for culturing intestinal stem cells and intestinal organoids (ENR medium)^2^ (Fig. 1a and Supplementary Fig. 2a,c). We wondered whether they would grow in conditions designed for gastric stem cells^3^. Shifting to medium conditions for stomach (Sto) organoids by using Wnt3a-conditioned medium (W), Fgf10 (f) and Gastrin (g) in addition to the ENR culture medium^3^ rescued the growth of *Cdx2*^*null*^ SI SCs and allowed them to form gastric-like organoids (Fig. 1a,b and Supplementary Fig. 2b,c), while control SI SCs formed intestinal organoids in the same medium. SC-derived *Cdx2*^*null*^ SI organoids cultured in stomach medium never generated Paneth cells, unlike their control SI organoids counterparts do (Fig. 1b).

### *Cdx2*^*null*^ SI organoids depend on gastric culture conditions

To rule out any impact of the culture conditions on the type of organoids generated by Sto and SI SCs, and on their differentiation marker expression, we analysed the transcriptome of organoids grown from wild-type stomach glands and small intestinal crypts by microarray. We show that they express a gastric and intestinal signature, respectively, regardless of whether they are cultured in stomach or intestinal conditions (Supplementary Fig. 3a). Hierarchical clustering on RNA-Seq analysis and data recovery for intestinal and stomach markers show that SC-derived SI organoids maintain their intestinal identity when grown in gastric medium (Supplementary Fig. 3b). The gene expression signature of both types of organoids is specific to the tissue of origin (Supplementary Fig. 3a,b). The stomach growth specificity of *Cdx2*^*null*^ SI organoids is therefore not the result of their culture in stomach conditions.

The inability of *Cdx2*^*null*^ SI SCs to grow and form organoids in intestinal conditions is not alleviated by a pulse of Wnt alone (Fig. 1a and Supplementary Fig. 2c), unlike it is the case for isolated wild-type SI SCs without Paneth cells^9^. The growth impairment of the *Cdx2*^*null*^ SI SCs in intestinal conditions *in vitro* is therefore not a mere consequence of their inability to generate Paneth cells, shown previously to reconstitute the niche of isolated SI SCs^9^. The growth of *Cdx2*^*null*^ SI SCs has become strictly dependent on Wnt, Fgf and Gastrin altogether, like the growth of control Sto SCs 3 (Fig. 1a and Supplementary Fig. 2c). This suggests a conversion of the *Cdx2*^*null*^ SI SCs into Sto SCs, and a change in identity and fate of the SI SCs by the loss of Cdx2. Failure to produce Paneth cells results from this identity change.

### *Cdx2*^*null*^ SI SCs lose intestinal and gain gastric markers

**Figure 2:**
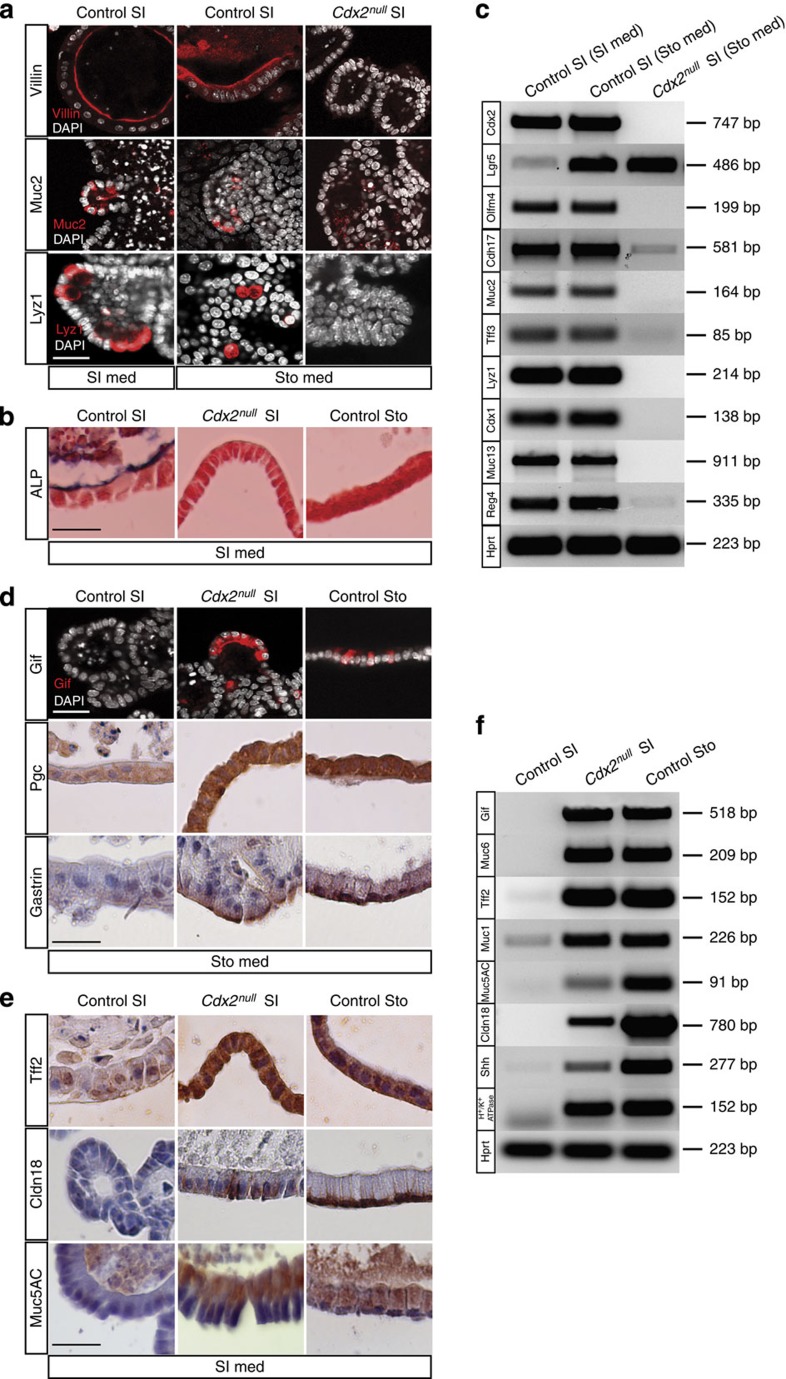
*Cdx2*^*null*^ SI organoids lost intestinal and express stomach markers. (**a**) *Cdx2*^*null*^ SI organoids grown from single SCs fail to express the intestinal markers Villin, Muc2 and Lyz1, whereas control SI organoids grown in the same stomach conditions (ENRWfg) do, like they do in intestinal (ENR) growth conditions. (**b**) *Cdx2*^*null*^ SI organoids and control Sto organoids grown for 10 days in intestinal culture medium do not express the intestinal differentiation marker ALP, whereas this marker is expressed in control SI organoids in the same growth conditions. Bar, 25 μm. (**c**) RT-PCR experiments using RNA from independent clones of control SI SC-derived organoids grown in intestinal and gastric conditions, and from *Cdx2*^*null*^ SI SC-derived organoids grown in gastric conditions; intestinal markers, *Olfm4, Cdh17, Muc2, Tff3, Lyz1, Cdx1, Muc13* and *Reg4.* (**d**) Organoids growing from single *Cdx2*^*null*^ SI SCs express the gastric markers Gif, Pgc and Gastrin, like control Sto organoids do, but unlike control SI organoids, all samples were grown in the same gastric conditions. (**e**) *Cdx2*^*null*^ SI organoids and control Sto organoids grown for 10 days in culture medium to allow gastric differentiation of control Sto organoids do express the gastric differentiation markers Tff2, Cldn18 and Muc5AC, Control SI organoids do not. Bar, 25 μm. (**f**) Expression detected by RT-PCR of the gastric markers *Gif*, *Muc6*, *Tff2, Muc1*, *Muc5AC, Claudin18, Shh* and *H*^*+*^*/K*^*+*^ *ATPase* in *Cdx2*^*null*^ SI organoids, compared with control SI and control Sto organoids. For *Gif*, *Muc6* and *Muc1* detection, the culture medium for all three samples was Sto medium; for *Tff2, Muc5AC, Claudin18, Shh* and *H*^*+*^*/K*^*+*^ *ATPase* detection, the medium used for all three samples was stomach differentiation medium allowing gastric differentiation of control Sto organoids; this medium corresponds to SI medium. med, medium. Images are representative of the results obtained in three independent experiments performed on each of two independent clones.

The panel of markers expressed by the organoids derived from *Cdx2*^*null*^ SI SCs grown in stomach conditions clearly reveals a gastric signature, unlike the panel of markers expressed by the intestinal controls in the same medium (Fig. 2a–f). As shown in Fig. 2a–c, ablation of Cdx2 led to strong downregulation of the intestinal stem cell markers *Olfactomedin4 (Olfm4)*^5^ and *Cadherin17 (Cdh17)*^10^, of the intestinal epithelial markers Villin^11^, *Cdx1* (refs 12, 13), *Mucin13 (Muc13)*^14^, *Reg4*^15^, and of markers of the different intestinal lineages, such as goblet cells (*Mucin2 (Muc2)*^16^,^17^,^18^,^19^,^20^ and *Trefoil factor 3 (Tff3*)^21^), Paneth cells (*Lysozyme1* (*Lyz1*))^9^ and enterocytes (apical Alkaline Phosphatase (ALP))^22^ (Fig. 2a–c). For all these markers, it is clear that they are expressed in control SI organoids, regardless of whether the culture conditions are the preferred conditions for intestinal or gastric organoids. In all cases these intestinal markers are not expressed in *Cdx2*^*null*^ SI organoids. Genes upregulated in *Cdx2*^*null*^ SI SC-derived organoids include markers of different gastric lineages, like *Gastric intrinsic factor (Gif)*^3^,^6^ and *PepsinogenC (Pgc)*^3^,^6^,^17^ in chief cells, *Mucin1 (Muc1)*^14^ and *Mucin5AC (Muc5AC)*^3^,^17^,^20^ in pit cells, *Mucin6 (Muc6)*^3^,^17^,^20^ and *Trefoil factor 2 (Tff2)*^3^,^6^,^21^ in neck cells, Gastrin (stomach-specific hormone)^3^ and *Claudin18* (*Cldn18*)^6^ in tight junctions of gastric epithelium, and *Sonic hedgehog (Shh)* and *H*^*+*^*/K*^*+*^ *ATPase*^6^ in parietal cells (Fig. 2d–f).

The conversion into gastric stem cells on loss of Cdx2 activity was not limited to the proximal SI SCs, known to reside in the digestive tract compartment abutting the stomach. *Cdx2*^*null*^ stem cells from proximal and distal small intestine both formed organoids that exhibited a gastric phenotype in terms of growth requirement and marker expression (Supplementary Fig. 4, showing downregulation of intestinal markers *Olfm4, Muc2*, *Lyz1* and *Cdx1*, and upregulation of gastric markers *Gif, Tff2, Muc6* and *Muc1* in *Cdx2*^*null*^ distal small intestine). For further analysis hereafter, we focus on the effect of inactivating Cdx2 in proximal SI SCs.

### Transcriptome analysis of *Cdx2*^*null*^ SI SCs

**Figure 3:**
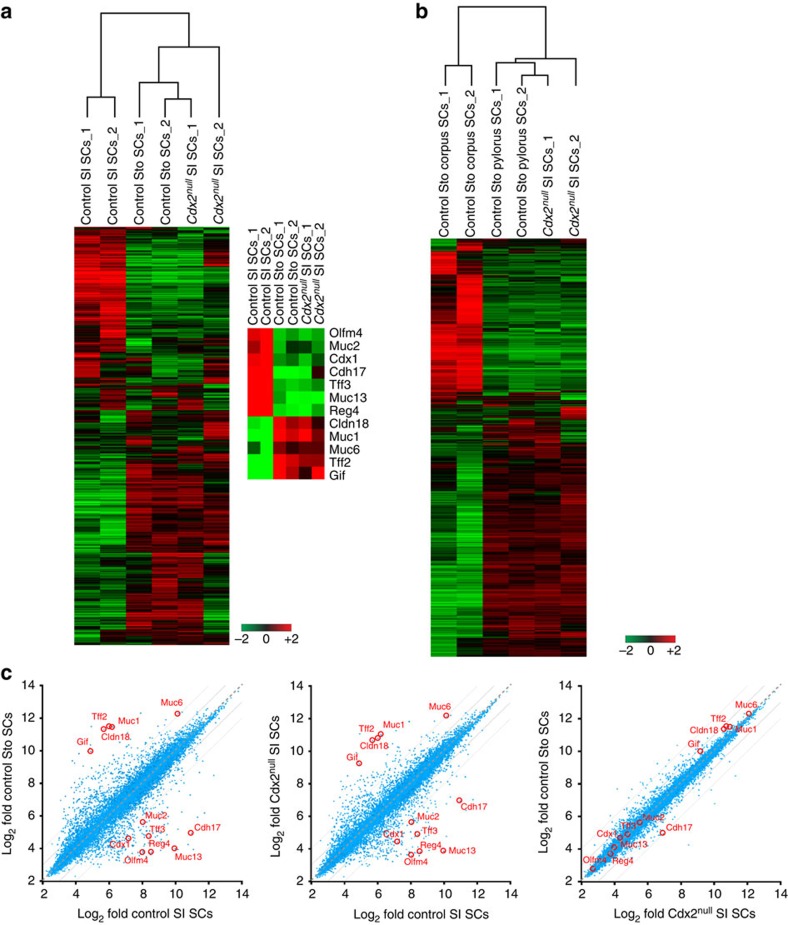
Transcriptome analysis of *Cdx2*^*null*^SI SCs. (**a**) Heatmap comparison of the transcriptome of *Cdx2*^*null*^ SI SCs with control SI SCs and Sto SCs (two independent samples of each) cultured in the same gastric conditions. Downregulated and upregulated genes considered to build the heatmap are the genes with at least a twofold change in transcription. Genes characterizing the signature of SI and Sto SCs are listed on the right of the heatmap: among the downregulated genes are the intestinal markers *Olfm4*, *Muc2, Cdx1*, *Cdh17, Tff3*, *Muc13 and Reg4*; among the upregulated genes are the gastric markers *Claudin18*, *Muc1, Muc6*, *Tff2* and *Gif*. Hierarchical clustering is shown on the top. (**b**) Transcriptome analysis of *Cdx2*^*null*^ SI SCs compared with control gastric pyloric and corpus SCs (two independent samples of each category of SCs). Hierarchical clustering is on the top. (**c**) Pair-wise scatter plot analysis of changes in gene expression (two independent samples each time) showing that *Cdx2*^*null*^ SI SCs exhibit much more gene expression similarity with control Sto SCs (graph on the right) than with control SI SCs (graph in the middle). Each dot (blue or red) in the graph represents a gene present on the array used in the Affymetrix analysis. Dots along the bisector line are similarly expressed in the two samples compared. Dots shown in red correspond to genes with higher expression (Log_2_ fold) in the sample indicated on the closest axis; the farther from the bisector, the bigger the difference. The genes the most altered in expression (red dots) correspond to the genes highlighted on the right of the heatmap in Fig. 3a.

In addition to testing a large number of markers specific to the small intestinal and gastric commitment of SC-derived organoids, we cultured organoids derived from single stem cells, FACS-sorted Lgr5-EGFP^hi^ stem cells again and collected RNA for microarray analysis (experimental scheme in Supplementary Fig. 5). This allowed us to determine the impact of the absence of Cdx2 in the SI stem cells themselves. Comparative transcriptome analysis confirmed that *Cdx2*^*null*^ SI SCs originating from SC-derived *Cdx2*^*null*^ SI organoids exhibit a gastric signature and cluster with the control stomach stem cells rather than with the control intestinal stem cells (Fig. 3a, two independent samples of each type of SCs). They express all stomach differentiation markers tested (*Cldn18*, *Muc1, Muc6, Tff2*, *Gif*, indicated on the right of the heatmap, Fig. 3a) at a higher level than SI SCs, while they have downregulated all small intestinal markers tested (*Olfm4*, *Muc2, Cdx1, Cdh17, Tff3*, *Muc13, Reg4*, Fig. 3a). We further strengthened this analysis by performing RNA-Seq on four additional independent samples of SC-derived *Cdx2*^*null*^ SI organoids, and four samples of control SI and Sto organoids. This independent transcriptome analysis confirmed that SC-derived *Cdx2*^*null*^ SI organoids are extremely similar to their stomach control (Supplementary Fig. 6a). The gene expression profile of the *Cdx2*^*null*^ SI SC samples established by microarray analysis (Fig. 3a) was compared with that of gastric corpus^23^ and pylorus SCs (Fig. 3b). This comparison made it obvious that *Cdx2*^*null*^SI SCs resemble pylorus SCs considerably and differ from corpus SCs. Taken together, these analyses clearly established that *Cdx2* inactivation has caused the intestinal stem cells to change their identity from intestinal to gastric pylorus. This change can be made visible at the genome-wide level, by establishing the pairwise comparison of transcriptional changes between *Cdx2*^*null*^ SI SCs and control Sto SCs (two independent samples of each), which shows much more similar transcriptional profiles than *Cdx2*^*null*^ SI SCs versus control SI SCs (Fig. 3c). The genes most altered in their expression between *Cdx2*^*null*^ SI SCs and control SI SCs (see highlighted genes on the right of the heatmap in Fig. 3a and middle graph in Fig. 3c) correspond to the set of markers that define the differences between control Sto and control SI SCs (Fig. 3c, graph on the left). These differences are absent when comparing *Cdx2*^*null*^ SI SCs and control Sto SCs (Fig. 3c, graph on the right). The changes in marker gene expression in *Cdx2*^*null*^ SI SCs have all been found to be significant in a quantitative analysis of the data from the microarray analyses (see text and Supplementary Fig. 6b where the projections on the *y*-axis reflect statistical significance). Examples of genes with highly significant changes for upregulated stomach markers are *Cldn18*, *Muc1, Tff2* and *Gif*, and for downregulated intestinal markers, *Olfm4*, *Muc2, Cdx1, Cdh17, Tff3*, *Muc13 and Reg4* (Fig. 3c and Supplementary Fig. 6b). These significant differences are not present between *Cdx2*^*null*^ SI SCs and control Sto SCs (graph on the right in Supplementary Fig. 6b). These pair-wise comparisons of the transcriptional changes between *Cdx2*^*null*^ SI SCs, and control Sto and SI SCs thus settle the much closer relationship between *Cdx2*^*null*^ SI SCs and Sto SCs.

**Figure 4:**
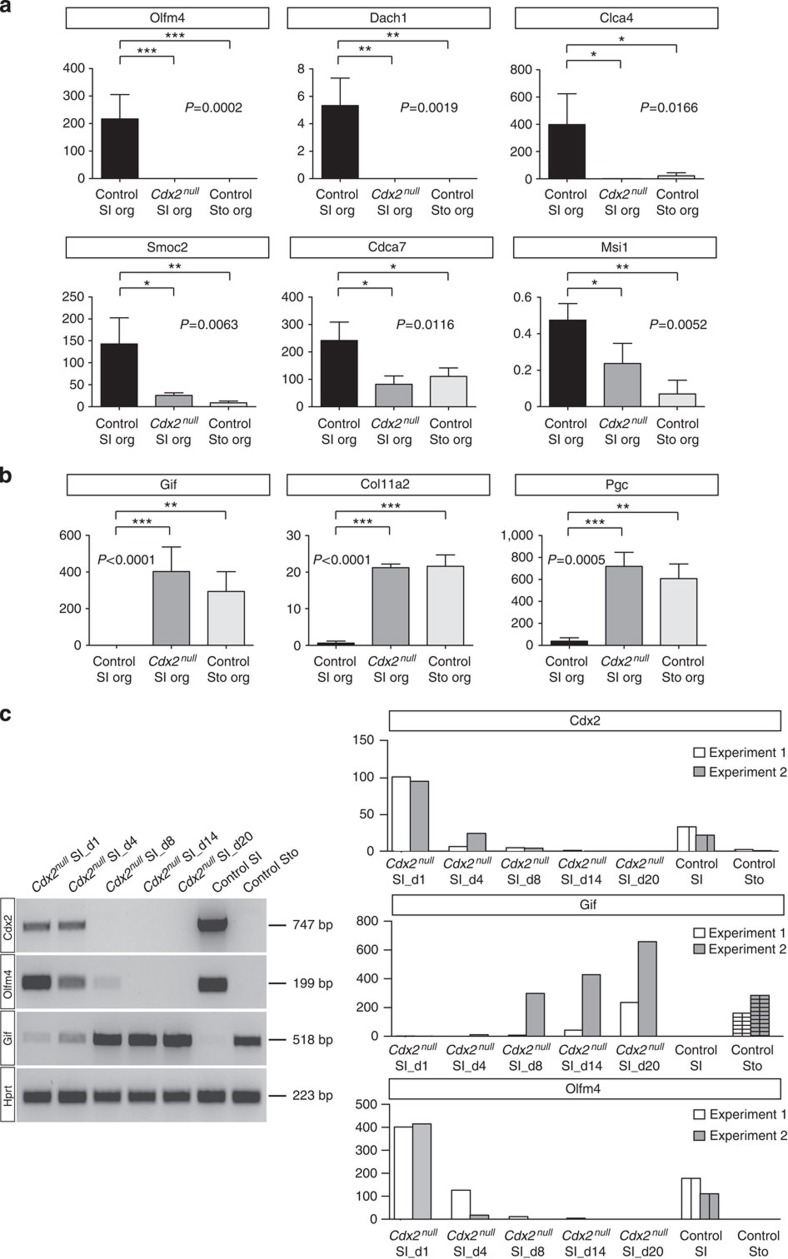
SI and Sto SC marker gene expression in *Cdx2*^*null*^ SI organoids. (**a**) Expression of the SI SC markers *Olfm4*, *Dach1, Clca4, Smoc2, Cdca7* and *Msi1.* org, organoids. (**b**) Expression of the stomach-specific SC markers *Gif*, *Col11a2, and PepsinogenC (Pgc)* in control SI organoids (black bar), *Cdx2*^*null*^ SI organoids (dark grey) and control Sto organoids (lighter grey). Along the *y*-axis, relative RNA amounts measured in three independent samples per condition, normalized for *Gapdh* expression. Error bars are s.d. values. For each marker, one-way analysis of variance according to the ANOVA test is indicated by the *P* value underneath the graph. *P*<0.05 indicates that the differences are significant. Tuckey’s test for multiple comparison was run for pair-wise comparison of the means of the samples, with *** meaning highly significant difference, ** very significant difference and * significant difference (see Methods for more details). org, organoids. (**c**) Time course of the downregulation of intestinal markers and the upregulation of gastric markers in SI SCs on inactivation of *Cdx2.* Left panel, RT-PCR for *Cdx2, Olfm4, Gif* and the house keeping gene *Hprt.* Right panel, graph representing the quantitative RT-PCR results of two independent time course experiments; light grey, five time points in experiment 1; dark grey, five time points in experiment 2; grey with internal patterns, control SI and control Sto.

Besides retrieving the expression levels of individual genes from the array data, we directly measured the transcriptional changes between *Cdx2*^*null*^ SI organoids and their stomach and intestinal controls in independent RNA samples isolated from organoid cultures developed from single SCs (three independent samples of each). We confirmed the loss (*Olfm4*, Fig. 4a), or strong decrease in expression of the intestinal stem cell markers *Dach1, Clca4, Smoc2, Cdca7* and *Msi1* (refs 4, 5), and the increase in expression of the stomach stem cell markers *Gif*, *Col11a2* and *Pgc*^3^ in *Cdx2*^*null*^ SI SCs compared with control SI SCs (Fig. 4a,b). This quantitative marker expression analysis (Fig. 4), together with the genome-wide transcriptome comparisons (Fig. 3 and Supplementary Fig. 6), indicate that these *Cdx2*^*null*^ SI SCs have genuinely converted to stomach pyloric stem cells.

### Cdx2 ablation rapidly converts SI SCs into pyloric SCs

Candidates to cause intestinal to gastric conversion following *Cdx2* ablation are Cdx2 direct targets previously established in other studies^10^,^12^,^13^,^15^,^16^,^17^,^18^,^19^, and clearly downregulated in the *Cdx2*^*null*^ SI SCs compared with control SI SCs, such as *Muc2* (refs 16, 17, 18, 19)*, Cdh17* (ref. 10), *Reg4* (ref. 15) and *Cdx1* (refs 12, 13) (Supplementary Fig. 7). The rapidity of the transcriptional response of genes to *Cdx2* inactivation could also indicate primary involvement of Cdx2 in this process. The kinetics of the *Cdx2*^*null*^ SI identity conversion into stomach was followed by measuring intestinal and gastric gene expression in organoids at different time points after *Cdx2* inactivation in SI SCs. *Cdx2* transcripts seem to have disappeared by day 8 (Fig. 4c). Expression of the SI SC marker *Olfm4* begins to decrease at that time and a strong upregulation of the stomach marker *Gif* is then already noticeable (Fig. 4c). These changes in the gene repertoire manifested on *Cdx2* inactivation therefore are quite rapid, occurring after a week of culture and preceding further passaging, in keeping with the hypothesis of a key role of Cdx2 in sustaining the intestinal programme.

### *Cdx2* expressing gastric organoids remain stomach-like

**Figure 5:**
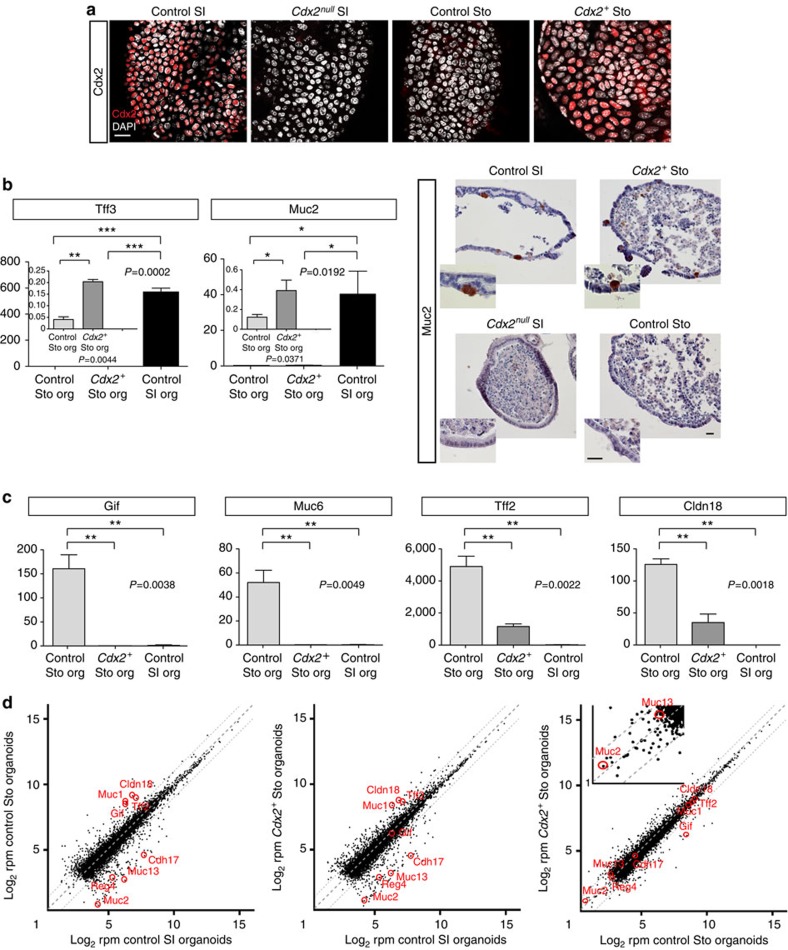
Expression of Cdx2 in wild-type Sto organoids fails to fully convert them into SI organoids. (**a**) Expression of Cdx2 in whole mounts of control SI organoids, *Cdx2*^*null*^ SI and control Sto organoids and *Cdx2* expressing Sto organoids (*Cdx2*^*+*^ Sto), all grown in Sto medium. Bar, 25 μm. (**b**) Left panel, quantitative measurement of transcripts of the intestinal markers *Tff3* and *Muc2* in *Cdx2*^*+*^ Sto compared with control Sto and SI organoids. The graph showing expression of intestinal markers in *Cdx2*^*+*^ Sto versus control Sto organoids is blown up in the insets to document the statistically significant small increase in expression (*t*-test, see Methods). For each marker, two independent experiments were performed, and the ANOVA and Tuckey’s tests were used (See Methods for more details). Asterisks in the inset were calculated using one tail *t*-test. org, organoids. Right panel, immunostaining for Muc2 in *Cdx2*^*+*^ Sto organoids, *Cdx2*^*null*^SI organoids and control SI and Sto organoids; a similar proportion of *Cdx2*^*+*^ Sto and control SI organoids (about 50%) contained positive cells for the immune reaction. Bar, 25 μm. (**c**) Quantitative measurement of transcripts (two independent experiments for each gene) of the gastric markers *Gif, Muc6, Tff2* and *Claudin18* in *Cdx2*^*+*^ Sto organoids compared with control Sto organoids and control SI organoids. Error bars, s.d. values. org, organoids. (**d**) Transcriptome analysis on performing RNA-Seq on *Cdx2*^*+*^ Sto organoids and control SI and Sto organoids. Scatter plot of the Log_2_ mean r.p.m. values per gene for *Cdx2*^*+*^ Sto against the Log_2_ mean r.p.m. values per gene for control organoids (four independent samples each time). The highlighted genes in the inset (graph on the right) are intestinal markers that are slightly upregulated (Log_2_ fold) in the *Cdx2*^*+*^ Sto compared with control Sto organoids. Dots along the bisector line are similarly expressed in the two samples compared. Outer dashed lines indicate the twofold difference boundary. Images in **a**, **b** (immunostainings) are representative of the results of three independent experiments each time.

We wondered whether Cdx2 could transform wild-type gastric epithelium in organoid cultures into tissues with intestinal identity. We generated a lentiviral stock expressing *Cdx2* from the PGK promoter (Supplementary Fig. 8) and infected dissociated wild-type pyloric stomach organoids in culture. After selection of infected cells, several clones were recovered, derived from stable infection of stem cells, which grew into organoids expressing Cdx2 (Fig. 5a) that could be passaged indefinitely. These organoids did not grow in intestinal medium. *Cdx2*-positive (*Cdx2*^*+*^) Sto organoids analysed after five passages in stomach culture conditions appeared to express the intestinal markers *Muc2* and *Tff3* at low level (Fig. 5b, left panel). In addition, immunohistochemistry revealed the expression of the Muc2 protein in a similar percentage of *Cdx2*^*+*^ Sto and control SI organoids, with the same type of epithelial distribution (Fig. 5b, right panel). Expression of these intestinal markers revealed a certain degree of intestinalization of the epithelium^19^,^22^,^24^,^25^. The *Cdx2*^*+*^ Sto organoids also downregulated stomach markers (*Gif, Muc6, Tff2* and *Cldn18*, Fig. 5c). Independent transcriptome analysis of *Cdx2*^*+*^ Sto organoids confirmed very mild changes in intestinal marker expression in *Cdx2*^*+*^ Sto (*Muc2* and *Muc13*) (Fig. 5d, graph on the right), but revealed that *Cdx2*^*+*^ Sto are globally more similar to control Sto than to control SI organoids (Fig. 5d). Expressing *Cdx2* is thus not sufficient to fully convert adult stomach stem cells into intestinal stem cells, unlike it is for maintaining the intestinal identity and fate of the small intestinal stem cells.

## Discussion

The role of Cdx2 in the adult intestinal epithelium had been suggested previously but could not thoroughly be investigated. Loss of function of *Cdx2* in the endoderm of the adult gastro-intestinal tract in mice *in vivo* was either examined at one time point^26^,^27^ or led to the generation of mosaic epithelium wherein wild-type crypts took over^6^. Generation of chimeras between wild-type and *Cdx2*^*null*^ intestines did not allow the examination of pure clonal populations of stem cells for prolonged periods of time^7^,^28^. We now demonstrate that the inactivation of a single transcription factor Cdx2 in the adult intestinal stem cells directly is sufficient to re-specify the identity and fate of these stem cells towards stem cells that exhibit a distinct committed programme: gastric stem cells. This re-specification is rapid and extensive, as the complete series of intestinal markers tested was downregulated while the complete series of gastric markers tested was turned on in the *Cdx2*^*null*^ SI SCs. This drastic re-specification occurs in the stem cells producing the endoderm epithelium *in vitro* in the absence of mesenchyme and is irreversible regardless of the culture conditions. We therefore conclude that Cdx2 is an absolute master controller of the intestinal character of the adult stem cells of the digestive tract epithelium in a cell-autonomous way.

## Methods

### Mice

All mice were in the C57Bl6j/CBA mixed background. The generation of the *Lgr5-EGFP-Ires-CreERT2* and *Cdx2*^*+/−*^ mice, as well as the protocols to genotype them, was described earlier, respectively, by ref. 1 and ref. 29. Generation and genotyping of the strain carrying the *Cdx2* conditional allele (*Cdx2*^*fl*^) were described by ref. 6. *Cdx2*^*−/fl*^*/Lgr5-EGFP-Ires-CreERT2* mice were generated by interbreeding *Lgr5-EGFP-Ires-CreERT2, Cdx2*^*fl/fl*^ and *Cdx2*^*+/−*^ mice. They have a normal phenotype. The animals used to isolate the intestine or stomach were males and females, without distinction. They were between 3 and 10 months old. All experiments using mice were performed in accordance with the institutional and national guidelines and regulations, under control of the Dutch Committee for Animals in Experiments, and under the licenses required in The Netherlands.

### Generating organoids from intestinal crypts and stomach glands

Isolation of small intestinal crypts and stomach pyloric glands, cell dissociation, cell culture and organoids formation and culture were adapted from those previously described by refs 2, 3. In brief, an isolated small intestine was opened longitudinally. It was washed with cold PBS and chopped into pieces of about 5 mm. Gastric glands were isolated from a dissected stomach. The stomach was opened along the greater curvature and washed with saline solution. The muscular layer of the stomach was removed and the remaining epithelium was divided into 5 mm pieces. The tissue fragments were then washed with cold PBS. Intestinal tissue fragments were incubated in 2 mM EDTA in PBS at 4 °C for 30 min. Stomach tissue fragments were incubated in 10 mM EDTA in PBS at 4 °C for 2–3 h. After removal of the EDTA solution, the tissue fragments were suspended in cold PBS with 10% Fetal Bovine Serum, using a 10-ml pipette. For the intestine, this suspension was the crypt fraction. It was passed through a 70-μm cell strainer (BD Bioscience). For the stomach, the suspension was the gland fraction and it was not filtered. Centrifugation of the suspensions at 200 *g* for 5 min allowed separating crypts or glands from single cells. The pellets enriched in crypts and glands were resuspended in Matrigel (BD Bioscience) and plated in 24-well plates (about 100 crypts or glands per 50 μl per well). After polymerization of the Matrigel, 500 μl of intestinal or gastric culture medium was added per well (Advanced DMEM/F12, (Invitrogen) containing growth factors: for intestinal medium, 10–50 ng ml^−1^ EGF (Invitrogen), R-spondin1 (conditioned medium) and Noggin (conditioned medium); for gastric medium, additional supplementation with 100 ng ml^−1^ FGF10 (Preprotech), Wnt3A (conditioned medium) and 10 nM Gastrin (Sigma-Aldrich)).

### Single stem cell sorting and organoid culture

To obtain *Cdx2*^*null*^ intestinal stem cells carrying the Lgr5-EGFP marker, 5- to 6-day- old small intestinal organoids generated from *Cdx2*^*−/fl*^*/Lgr5-EGFP-Ires-CreERT2* mice were incubated with 1 μM of 4-hydroxytamoxifen in intestinal culture medium^2^,^8^ for 16 h to activate the Cre recombinase. Controls were 4-hydroxytamoxifen-untreated small intestinal (Control SI) and stomach (Control Sto) organoids issued from mice with the same genotype. The organoids were dissociated and sorted for EGFP^hi^ by using MoFlo cell sorter (DAKO) as described by ref. 3 with some modifications. In brief, intestinal or stomach organoids were dissociated in culture medium by trituration with a glass pipette, followed by trypsinization in TrypLE Express (GIBCO). After incubation at 37 °C for 5–10 min, cells were resupended in Advanced DMEM/F12 and spun down. Pellets were resuspended in stomach medium (ENRWfg) supplemented with 10 μM Rock inhibitor Y-27632 (Sigma-Aldrich) and 0.8 Units μl^−1^ DNAse for culture purposes. The suspension was passed through a 40 μM mesh filter. EGFP^hi^ cells were sorted by flow cytometry (MoFlo; DAKO). Single viable (negative staining for propidium iodide) epithelial cells were gated by forward scatter, side scatter and pulse-width parameter. Sorted cells were collected in culture medium and embedded in Matrigel (BD Bioscience) at one cell per well (in 96-well plates, 5 μl Matrigel per well). Intestinal or stomach culture medium (250 μl for 48-well plates, 100 μl for 96-well plates) containing the ROCK inhibitor Y-27632 (10 μM) was overlaid.

Control SI SCs grew and could be passaged indefinitely in small intestinal medium (SI med, ENR^2^) or in stomach medium (Sto med, ENRWfg^3^). *Cdx2*^*null*^ SI and control Sto SCs were cultured in stomach medium^3^. Medium was refreshed every 2 days. Passage of the generated organoids was performed in split ratios of 1:4 and subsequently about once per week. Growth of the organoids and expression of Lgr5-EGFP were documented by using an EVOS fl (AMG) microscope.

Fifty-nine independent clones of *Cdx2*^*null*^ SI SCs were generated during these experiments, and the analysis of 15 of these is documented in detail in this work. An equivalent number of clones of both types of controls (small intestinal and stomach) was analysed.

### Genotyping

*Cdx2*^*null*^ and control stem cells sorted for EGFP^hi^ by flow cytometry were genotyped on using 1,000 cells. Organoids were genotyped after collecting them from the clonal cultures. This procedure was repeated several times during the culture to rule out that organoids grew from cells that had escaped 4-hydroxytamoxifen-induced *Cdx2* inactivation (see Supplementary Fig. 1). Primers used are listed in Supplementary Materials.

### Gene expression analysis by RT-PCR

For RT-PCR analysis, RNA was extracted from *Cdx2*^*null*^ SI, control SI and control Sto organoids using the RNeasy Mini RNA Extraction Kit (Qiagen) and reverse-transcribed using Moloney Murine Leukemia Virus reverse transcriptase (Promega). cDNA was amplified in a thermal cycler (Veriti 96 well Thermal Cycler, Applied Biosystems, London, UK). Primers used are listed in Supplementary Materials. For full gel panels see Supplementary Figs 9 and 10.

For real-time quantitative PCR analysis, cDNA was amplified with iQSyberGreenSupermix (Biorad) on a Biorad CFX Connect Real-Time PCR System. Data were analysed using Biorad CFX Manager Software Version 2.1. Gene expression was normalized according to the expression of the housekeeping gene *Gapdh*. The primers used are listed in Supplementary Materials.

### Statistical analysis

Statistical analysis was performed on Graphpad Prism Software Version 5.0. Results are expressed as mean±s.d. One-way analysis of variance (ANOVA) was used to evaluate the differences between group means. Student’s *t*-test was used to analyse the difference between the means of two samples. *P*=0.05 was taken as the maximum value for significance. Tuckey’s Multiple comparison test was used to find whether pairwise comparison of means are significantly different from each other. The level of significance is represented by asterisks (***, highly significant *P*<0.0001; **, very significant *P*<0.001; *, significant *P*<0.05).

### Immunostaining for intestinal and stomach markers

For immunofluorescent staining on whole mounts, samples were fixed with 4% PFA for 20 min at room temperature, permeabilized with PBS 0.5% Triton-X100- 1% BSA and incubated overnight with the primary antibodies. Following several washes in PBS with 0.3% Triton-X100- 0.05% BSA, samples were incubated with the secondary antibody. Primary antibodies were mouse anti-Villin (1:100, Santa Cruz), rabbit anti-Muc2 (1:1,000, Santa Cruz), rabbit anti-Lysozyme (1:1500, DAKO), rabbit anti-Gastric Intrinsic Factor (1:24,000, generous gift from David Alpers) and mouse anti-Cdx2 (1:1,000 Biogenex). Secondary antibodies used were Alexa Fluor 568 donkey anti-mouse IgG (H+L) (Invitrogen), Alexa Fluor 488 donkey anti-mouse IgG (H+L) (Invitrogen) and Alexa Fluor 568 donkey anti-rabbit (Life Technologies). Nuclei were stained with DAPI (Invitrogen). Images of the organoids were acquired using Leica TCS SPE and TCS SPE Live confocal microscopes. Images were analysed using the Leica LAS AF Lite software.

For immunostainings on sections, small intestinal and stomach organoids were fixed and embedded in paraffin, and staining was performed on 4 μm sections according to standard protocols. Primary antibodies were mouse anti-Muc5AC (1:200, Novocastra), rabbit anti-Gastrin (1:500, Novocastra), rabbit anti-Claudin18 (1:800, Invitrogen), rabbit anti-Muc2 (1:1,000, Santa Cruz), goat anti-Tff2 (1:300, Santa Cruz), sheep anti-PepsinogenC (1:50,000, Abcam). The peroxidase-conjugated secondary antibodies used were Mouse EnVision+ (DAKO), BrightVision poly HRP-anti-rabbit IgG (Immunologic), Southern Biotech Rabbit anti-goat IgG (HIL)-UNLB and polyclonal rabbit anti-sheep immunoglobulins/HRP (DAKO). Images of the organoids were acquired using Leica DFC500 camera and Nikon Eclipse E600 microscope.

### Cell sorting and RNA isolation for microarray analysis

Organoids were dissociated and sorted by flow cytometry (MoFlo, DAKO) as described above^3^. Several independent samples were analysed for each condition. EGFP^hi^ stem cells were collected in Trizol LS (Invitrogen) and RNA isolated by using RNeasy Micro RNA Extraction Kit (Qiagen). RNA concentration and quality was determined using a NanoDrop (NanoDrop Technologies, Wilmington, DE, USA) and Agilent 2100 Bioanalyzer (Agilent Technologies, Palo Alto, CA, USA), respectively. Fragmentation of cRNA, hybridization to genome-wide mRNA expression platform harbouring 20,819 unique genes (Affymetrix mouse gene ST1.1 Array Plate) and scanning were carried out according to the manufacturer’s protocol (Affymetrix, Santa Barbara, CA, USA) at the MicroArray Department of the AMC (Amsterdam, the Netherlands). Note that the array used four probe sets for *Cdx2*, covering the three exons of the gene, thus including the part of the gene that is still present in the *Cdx2*^*null*^ SI SCs. The expression data extracted from the raw files were normalized with the RMA-sketch algorithm from Affymetrix Power Tools. Pair-wise scatter plot and volcano plot were generated by using the R2 web application, which is freely available at http://r2.amc.nl ‘Microarray analysis and visualization platform’. False discovery rate (FDR) statistical method was used to correct for multiple comparisons test. An expression difference of twofold (Log_2_ fold) and a level of significance of *P*<0.05 (*t*-test) was defined as a threshold for significantly changed genes. Cluster analysis was performed by using Cluster 3.0 and Tree View 1.60 softwares. An expression difference of twofold (Log_2_ fold) was defined as a threshold for significantly changed genes. Microarray data have been deposited in Gene Expression Omnibus (accession number GSE51751).

### RNA extraction from organoids and RNA-Seq

Organoids were freed from Matrigel and resuspended in RLT buffer (Qiagen). RNA was extracted from *Cdx2*^*null*^ SI, Control SI and Control Sto organoids using the RNeasy Mini RNA Extraction Kit (Qiagen). For mRNA sequencing, 10 ng of total RNA was used as starting material. The RNA was processed using the CEL-Seq protocol^30^ and sequenced on an Illumina Nextseq using 75 bp paired end sequencing. After sequencing read 1 was aligned with the mm10 RefSeq mouse transcriptome downloaded from the UCSC genome browser^31^ using bwa^32^ with default parameters. Read 2 contains a barcode identifying the sample from which the read originated. CEL-Seq only sequences the most 3′ end of a transcript and generates one read per transcript. Samples were r.p.m. (reads per million) normalized. Only genes with more than 10 r.p.m. in at least four samples were used for subsequent analysis. Sequencing data have been deposited in Gene Expression Omnibus under accession number GSE62784.

### *Cdx2* ectopic expression in wild-type stomach organoids

Full-length *Cdx2* cDNA was cloned behind the PGK promoter into Sal1 and Xho1 restriction sites of the pLV_pGK_2A_dsRED_IRES-Puro plasmid (generous gift from Henner Farin from the Clevers lab) using the following primers:

forward, 5′- GAACTAAACCGTCGACGCCACCATGTACGTGAGCTACCTT -3′;

reverse, 5′- CGCTTCCGGACTCGAGCTGGGTGACAGTGGAGTTTAAAAC -3′.

To generate a lentiviral stock expressing *Cdx2*, the Human Embryonic Kidney 293T (HEK 293T) cells were cultured in 10% FCS DMEM in a 150 cm dish, to a confluency of about 80%. Cells were transiently transfected with the *Cdx2* expressing vector (30 μg) in the presence of polyethylenimine (PEI) (Polysciences) in serum-free DMEM, according to the manufacturer’s instructions. The medium was changed once on the next day to remove PEI. After two more days, the medium was collected, passed through a 0.45-μm filter and centrifuged it at 8,000 *g* overnight at 4 °C. The supernatant was discarded and the pellet resuspended in 500 μl of infection medium (stomach medium plus 10 mM nicotinamide (Sigma-Aldrich) plus 10 μM Y27632 (p160 ROCK inhibitor, Sigma-Aldrich) plus 8 μg ml^−1^ Polybrene (Sigma-Aldrich).

Stomach organoids were infected by a lentiviral suspension and cultured under puromycin selection according to the protocol previously described by ref. 8 modified as follows. In brief, about 500 organoids were transferred to a 15-ml Falcon tube and dissociated with a fire-polished glass pipette. Organoid fragments were incubated with TripLE Express (GIBCO) for 5 min at 37 °C. Medium containing 5% serum was added, and the cells were pelleted at 400 *g* for 5 min. The supernatant was discarded and the cell clusters resuspended in 500 μl of infection medium (see above). Cell clusters were combined with 500 μl of viral suspension and transferred into four wells of a 48-well culture plate. The plate was centrifuged at 600 *g* at 32 °C for 60 min and placed for another 6 h in an incubator at 37 °C. After this time, the cells were collected, transferred into 1.5 ml Eppendorf tube and spun them down at 400 *g* for 5 min. The supernatant was discarded, and the pellet was resuspended in 100 μl Matrigel (BD Biosciences) and distributed to two wells of a 24-well culture plate. 500 μl of infection medium without polybrene was added per well. Two days after infection, the medium was refreshed and puromycin (2 μg ml^−1^) was added. Organoids were maintained in Sto culture conditions and passaged every week. Marker dsRed expression was scored and Cdx2 protein expression assayed by immunofluorescence.

## Author contributions

J.D., S.S. and M.B. designed the experimental approach; S.S., M.B., M.H., L.K. and M.vd W. designed and performed the experiments; T.S. contributed to the initial discovery of the *Cdx2*^*null*^ intestinal stem cell properties; F.B. participated in the generation of the *Cdx2* conditional mouse mutant line; A.v.O. supervised the RNA sequencing work; H.C. provided the expertise and facilities to perform the work with organoid cultures; J.D. and S.S. wrote the manuscript.

## Additional information

**How to cite this article:** Simmini, S. *et al*. Transformation of intestinal stem cells into gastric stem cells on loss of transcription factor Cdx2. *Nat. Commun.* 5:5728 doi: 10.1038/ncomms6728 (2014).

**Accession codes:** Accession number for the microarray and RNA-Seq data are respectively GSE51751 and GSE62784.

## Supplementary Material

Supplementary Information Supplementary Figures 1-10 and Supplementary Methods
