# Supplementary material for: Transformation of intestinal stem cells into gastric stem cells on loss of transcription factor Cdx2
Source: Nat Commun. 2014 Dec 11;5:5728. doi: 10.1038/ncomms6728 (PMC4284662; doi:10.1038/ncomms6728)
Supplement: Supplementary Information — Supplementary Figures 1-10 and Supplementary Methods [file ncomms6728-s1.pdf]

Supplementary Figures

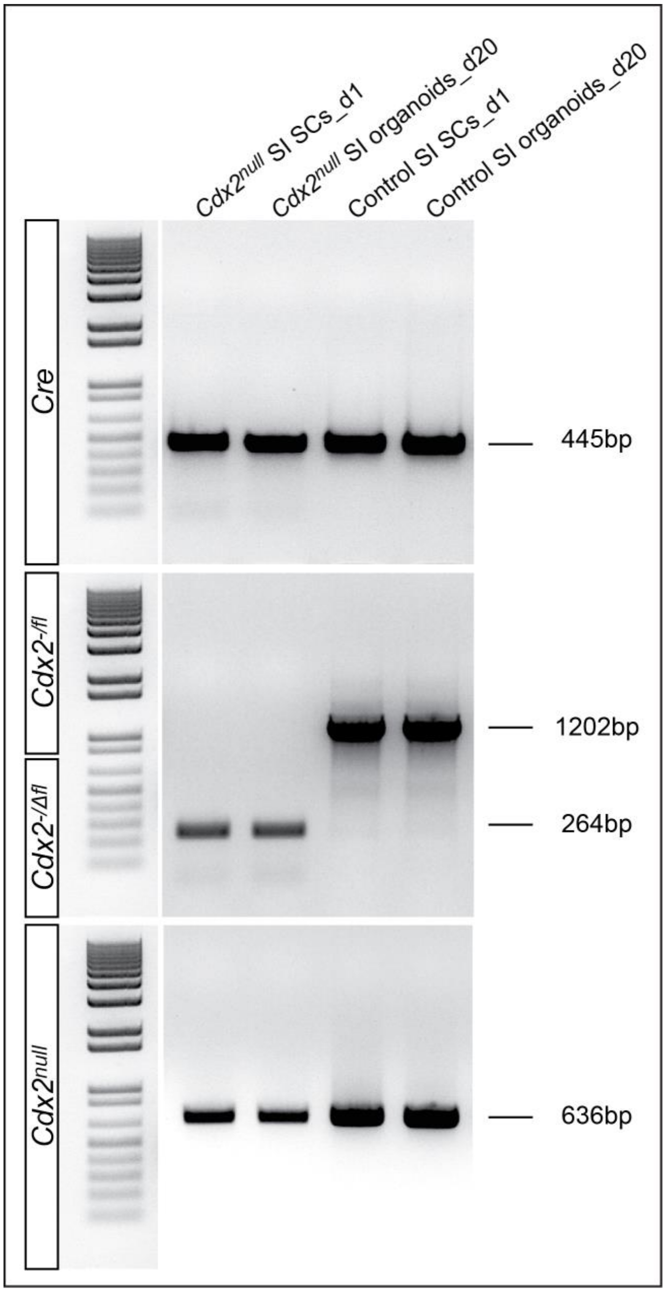

Supplementary Fig. 1. Genotyping of *Cdx2^{null}* and control SI SCs at different time points of the cultures. Shown here are the diagnostic PCR results for FACS-sorted *Cdx2$^{-/fl}$/Lgr5-EGFP-Ires-CreERT2* SI SC samples after treatment with 4-hydroxytamoxifen (*Cdx2^{null}*) or untreated (control). The *Cre* allele and one *Cdx2^{null}* allele are present everywhere, the *Cdx2^{fl}* allele is present solely in the absence of 4-hydroxytamoxifen (control), and the inactivated *Cdx2$^{\Delta fl}$* allele is present solely in the presence of 4-hydroxytamoxifen (*Cdx2^{null}*). Genotyping was performed on samples of clonal cultures one day after 4-hydroxytamoxifen treatment and repeated after two passages to verify homogeneity of the *Cdx2^{null}* organoids.

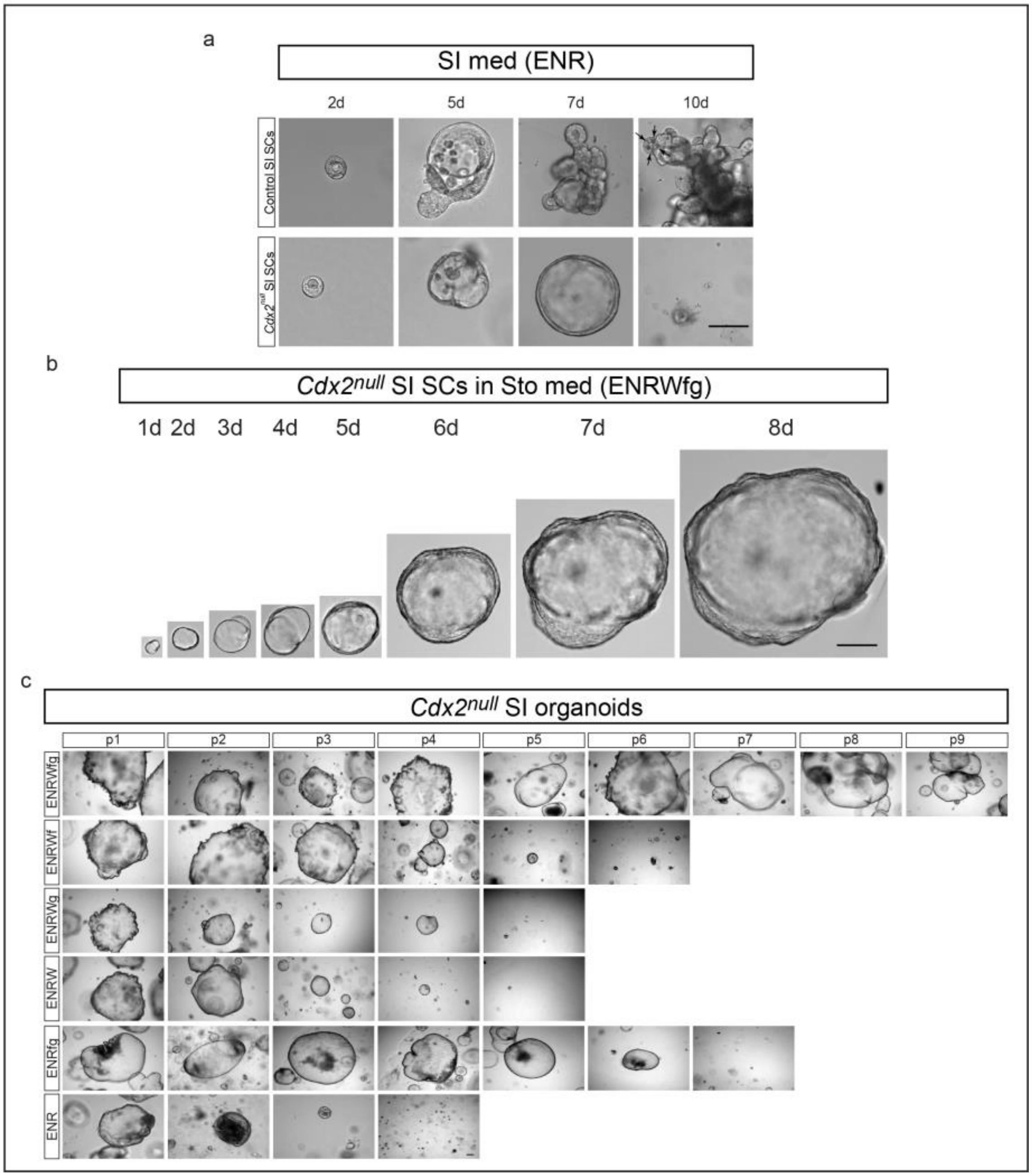

Supplementary Fig. 2. $Cdx2^{null}$ SI SCs require stomach growth conditions and generate stomach organoids. a. $Cdx2^{null}$ SI SCs fail to generate buds and instead become cystic and die in intestinal medium ENR, whereas their control counterpart grow, generate numerous buds and can be passaged indefinitely. Arrows indicate Paneth cells. **b.** Isolated $Cdx2^{null}$ SI SCs grow as gastric organoids in stomach medium, ENRWfg, containing ENR supplemented with Wnt3a (W), Fgf10 (f) and Gastrin (g). **c.** $Cdx2^{null}$ small intestinal organoids totally depend on the stomach-specific organoid conditions to grow. Views of a growing culture of SC-derived $Cdx2^{null}$ SI organoids, in stomach medium ENRWfg (first row); the first 9 of a large number of passages are shown. The following rows show the evolution of such cultures when deprived of, respectively Gastrin (second row), Fgf10 (third row), Fgf10 and Gastrin (fourth row), Wnt3a (fifth row), and Wnt3a, Fgf10 and Gastrin (last row). The latter condition (ENR or SI medium) does not allow the culture to be passaged more than once or twice. Bar, 150 µm. med, medium.

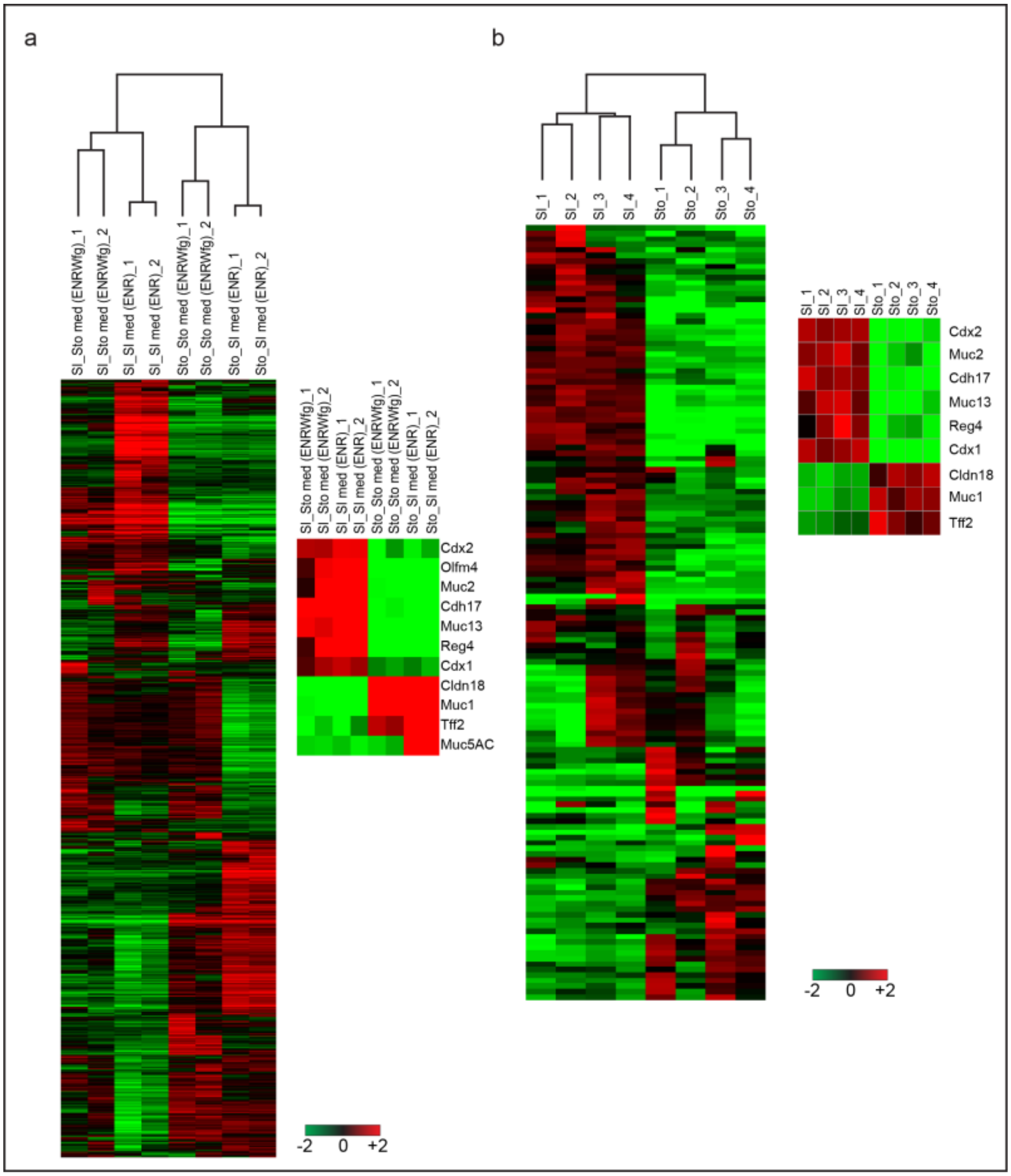

Supplementary Fig. 3. Organoids express an organ-specific signature independently of the culture conditions. a. Comparison of the transcriptional profiles by microarray analysis of two independent replicates of SI and Sto samples cultured in either SI or Sto conditions. Hierarchical clustering shows that the SI samples separate from Sto samples no matter in which medium they are cultured. Heatmap with specific markers on the right shows the expression of intestinal and gastric markers relevant for SCs identity and confirms that the culture conditions do not alter the intestinal or gastric signature of these SCs. Intestinal markers, *Cdx2, Olfm4, Muc2, Cdh17, Muc13, Reg4* and *Cdx1*; gastric markers, *Cldn18, Muc1, Tff2* and *Muc5AC*. **b.** mRNA sequencing data showing Log_2 fold changes in gene expression in Sto and SI organoids grown in the same stomach medium (4 independent samples), compared to the mean across all samples in the analysis. Genes with at least a two-fold change compared to the mean are included in the heatmap. For scaling purposes genes that have more than four-fold change are depicted as four-fold change. Inset on the right shows a selection of marker genes for intestine (*Cdx2, Muc2, Cdh17, Muc13, Reg4* and *Cdx1*) or stomach (*Cldn18, Muc1* and *Tff2*). med, medium.

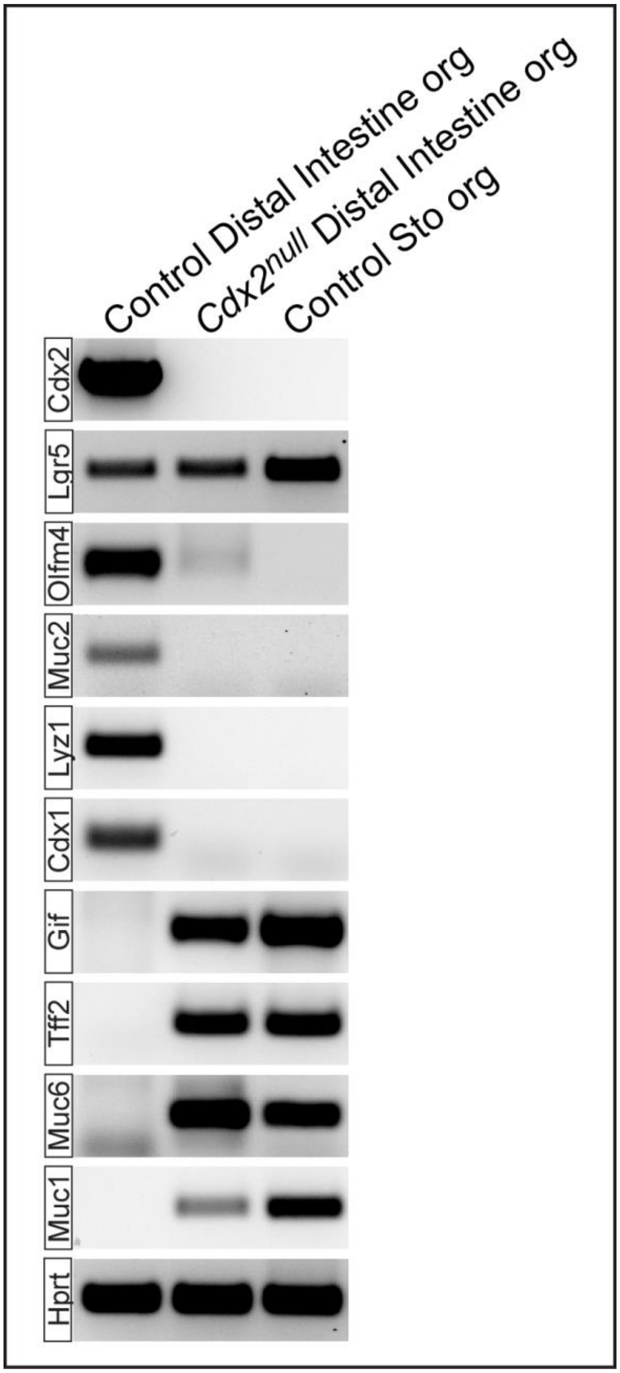

Supplementary Fig. 4. SCs from the distal small intestine also convert to Sto SCs in the absence of Cdx2. RT PCR experiments using RNA from SC-derived control distal intestinal organoids (first column), *Cdx2*^{null} distal intestinal organoids (second column) and control Sto organoids (third column), all grown in gastric conditions; intestinal markers *Olfm4, Muc2, Lyz1,* and *Cdx1,* are expressed in control distal intestinal organoids and fail to be expressed in *Cdx2*^{null} distal intestinal organoids; gastric markers *Gif*, *Tff2, Muc6*, and *Muc1* are not expressed in distal intestinal organoids but strongly upregulated in *Cdx2*^{null} distal intestinal organoids. This experiment was performed three times using independently generated samples. org, organoids.

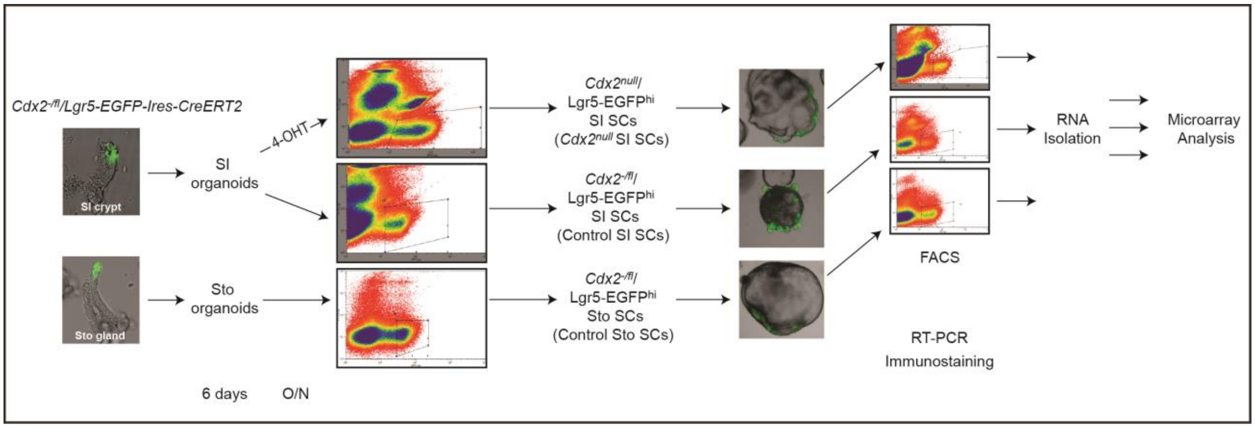

Supplementary Fig. 5. Structure of the experiments to characterize single clones of *Cdx2^{null}* SI SCs versus controls. Each step of the protocol is described in the Methods section (Generating organoids from intestinal crypts and stomach glands, Single cell sorting and organoid culture, and Cell sorting and RNA isolation for microarray analysis).

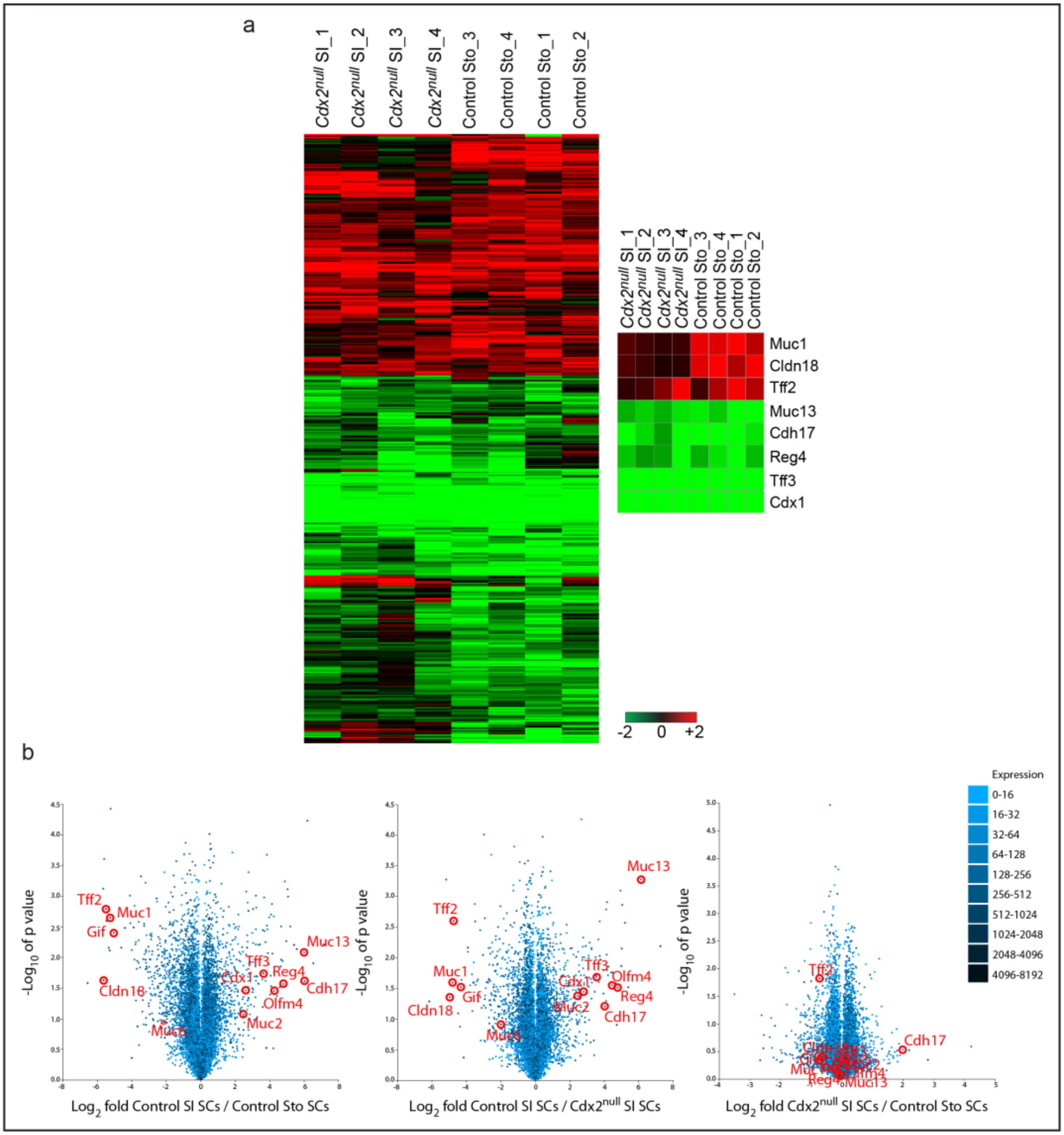

Supplementary Fig. 6. a. Transcription profile of additional SC-derived *Cdx2*^{null} SI versus Sto control organoids. mRNA sequencing data comparing Log_2 fold changes in SC-derived *Cdx2*^{null} SI and control Sto organoids (4 independent samples of each), each relatively to control SI organoids (mean of 4 independent samples). Genes with at least an average of two-fold change compared to the control SI organoids are included in the heatmap. For scaling purposes genes that have a more than four-fold change are depicted as four-fold change. Extraction of data corresponding to stomach markers (*Muc1, Cldn18* and *Tff2*) intestinal (*Muc13, Cdh17*, *Reg4*, *Tff3* and *Cdx1*) and is shown on the right. The comparison stresses the similarity between *Cdx2*^{null} SI and control Sto organoids. **b. Quantitative statistical analysis of the transcription changes measured by microarray between *Cdx2*^{null} SI SCs and controls.** Pairwise volcano plot analysis of the microarray data shown in Fig. 3a (two independent

samples of each type of SCs) shows the differences, using pair-wise comparisons, between *Cdx2^null^* SI SCs, control Sto SCs and control SI SCs. Each gene present on the Affimetrix array used is represented by a dot in the graphs, with darker color meaning higher expression level. In each graph, genes projecting on the x value zero do not show change in expression in the two samples compared. Per graph, genes indicated in red projecting on negative values on the x axis are decreased in expression in the first sample of the comparison versus the second, and genes indicated in red projecting on positive values of the x axis are increased in expression in the first sample of that comparison. Projections on the y axis indicate statistical significance ($-Log_{10}$ of p value). The genes highlighted in red are the same markers as highlighted in the heatmap of Fig. 3a, and this marker gene series overlaps with the gene series studied by independent direct quantitative RNA analysis (Fig. 4 and Supplementary Fig. 7).

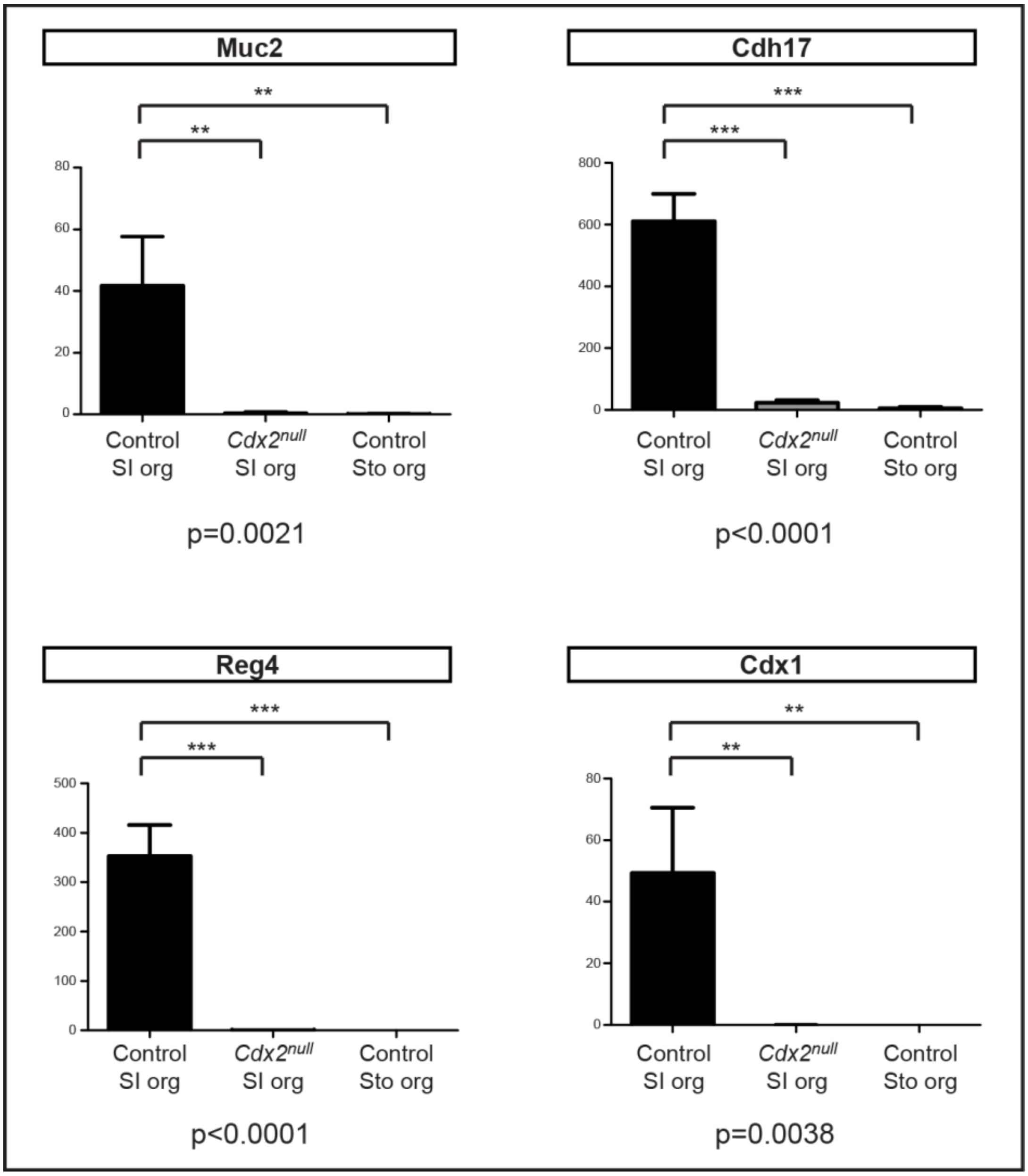

Supplementary Fig. 7. Assessment of the down regulation of Cdx2 targets in independent samples of *Cdx2^{null}* SI versus controls. Direct Cdx2 targets normally expressed in control SI SCs such as *Muc2, Cdh17*, *Reg4* and *Cdx1* were considerably downregulated in the *Cdx2^{null}* SI organoids compared with control SI organoids. These markers are lowly and similarly expressed in *Cdx2^{null}* SI and in control Sto organoids. Quantitative RT PCR analysis was performed on three independent samples of each type of material. Values along the y axis are relative amounts of RNA normalized for *Gapdh* expression. Error bars are standard deviations. For each marker, one way analysis of variance according to the ANOVA test is indicated by the p value underneath the graph. $p<0.05$ indicates that the differences are significant. Tuckey's test for multiple comparison was run for pair-wise comparison of the means of the samples, with *** meaning highly significant difference, ** very significant difference and * significant difference. See Methods for more details about statistical analysis. org, organoids.

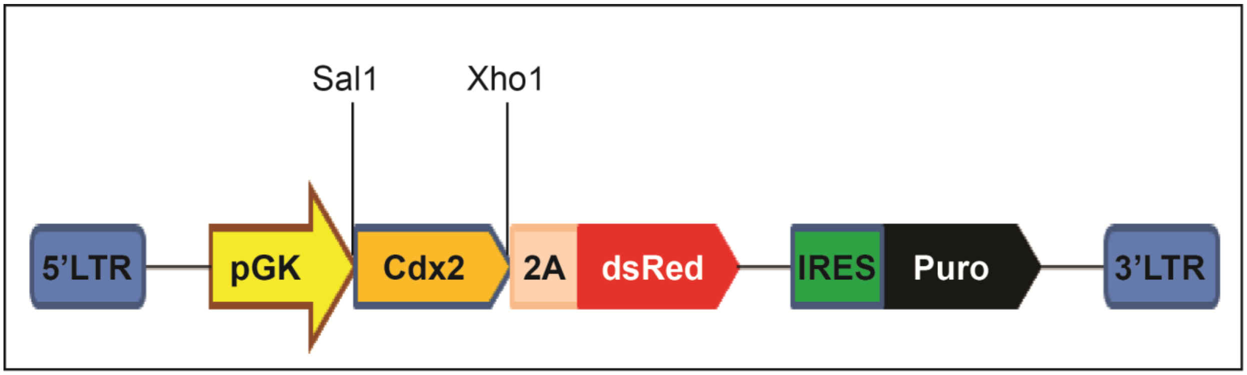

Supplementary Fig. 8. Lentiviral expression construct used to force Cdx2 expression in Sto organoids. Full-length *Cdx2* cDNA was cloned behind the PGK promoter into Sal1 and Xho1 restriction sites of the pLV_pGK_2A_dsRED_IRES-Puro plasmid.

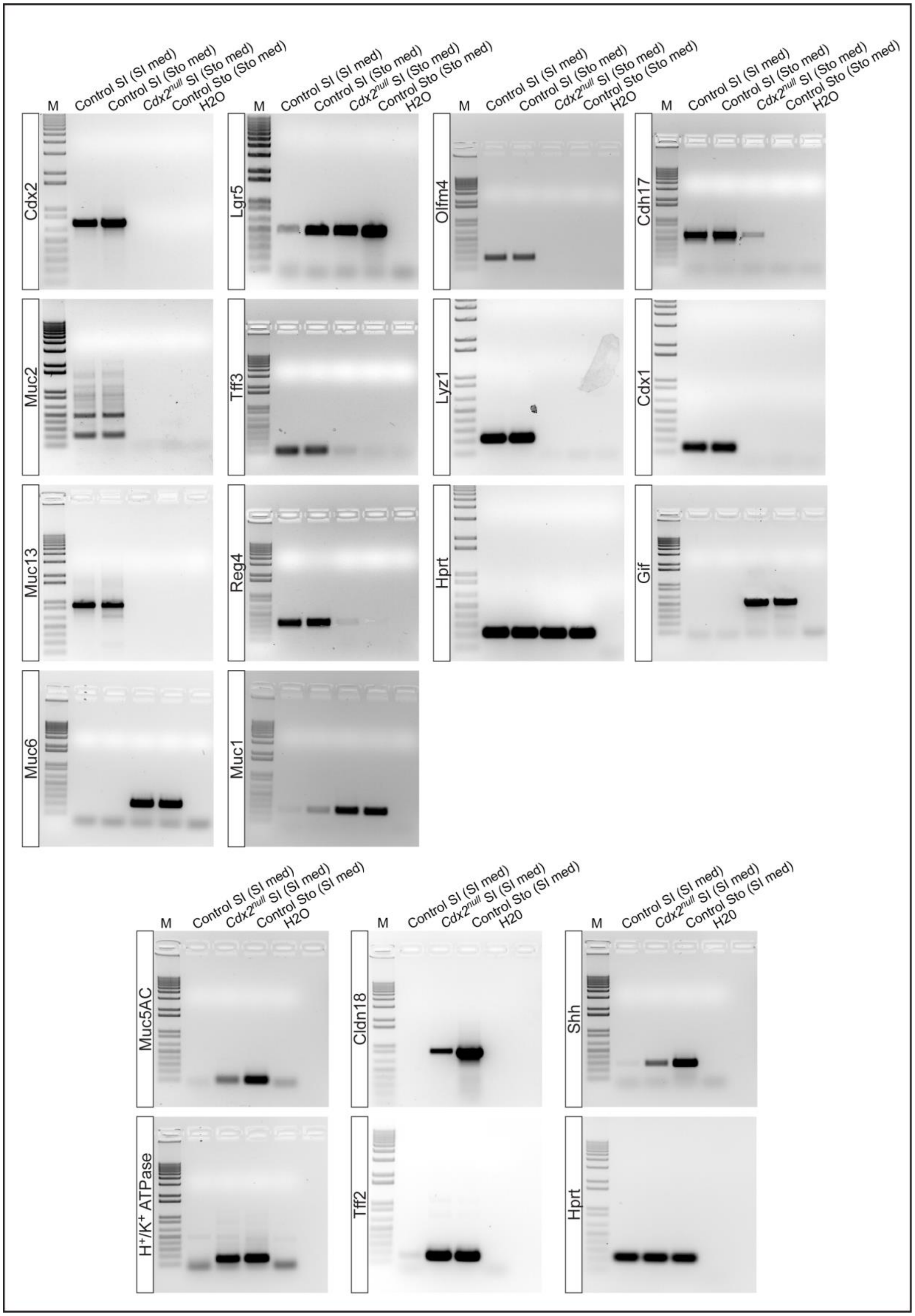

Supplementary Fig. 9. Full gels corresponding to the data shown in Fig, 2c and f.

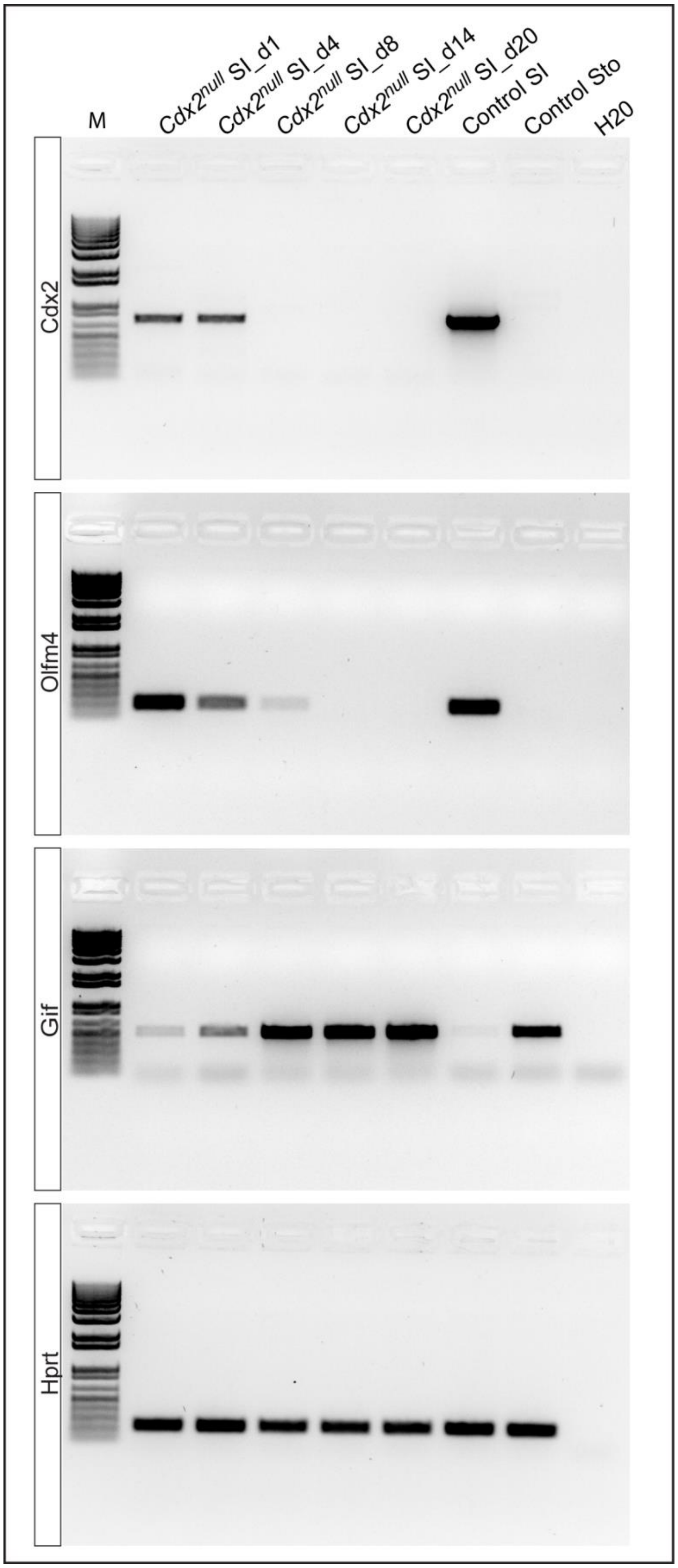

Supplementary Fig. 10. Full gels corresponding to the data shown in Fig, 4c left panel.

Supplementary Methods

Primers for genotyping

Primer	Sequence
Cre-For	CCGGGCTGCCACGACCAA
Cre-Rev	GGCGCGGCAACACCATTTTT
Cdx2^{fl}-For	TGGGGCAATCTTAATGGGTA
Cdx2^{fl}-Rev	TGTAGCCTCGACTTGGCTTT
Cdx2$^{\Delta fl}$-For	ACGCGTTCCAAGTGAAAGGA
Cdx2$^{\Delta fl}$-Rev	CGCTCTTTCTCTGTCCAAGTG
Cdx2^{null}-For	ATATTGCTGAAGAGCTTGGCGGC
Cdx2^{null}-For	TAAAAGTCAACTGTGTTCGGATCC

Primers for RT PCR

Primer	Sequence
Cdx2-For	GTACACAGACCATCAGCGGC
Cdx2-Rev	CCACCCCATCCAGTCTCACT
Lgr5-For	TGCCATCTGCTTACCAGTGTTGT
Lgr5-Rev	ATTCCGTCTTCCCACCACGC
Olfm4-For	GCCACTTTCCAATTTCAC
Olfm4-Rev	GAGCCTCTTCTCATACAC
Cdh17-For	CAAGTCTGTGCACCAAGCAC
Cdh17-Rev	TGCCATAGCCAAGTTGGAGG
Muc2-For	GAACGGGGCCATGGTCAGCA
Muc2-Rev	CATAATTGGTCTGCATGCC
Lyz1-For	GAGAACCGAAGCACCGACTATG
Lyz1-Rev	CGGTTTTGACATTGTGTTCGC
Cdx1-For	GCTCACAGAGCGGCAGGTAA
Cdx1-Rev	GGTGTGGGAGTGCCATCCAG
Muc13-For	AAAACTCCAGGCAGCGAAGG
Muc13-Rev	AATGTCCCCAGGGAATGTCG
Reg4-For	GCCTTGCACAAACTCTTCCC
Reg4-Rev	TGGTTTGACAGAGACGCAGT
Tff3-For	TTGCTGGGTCCTCTGGGATA
Tff3-Rev	GACATTTGCCGGCACCATAC
Gif-For	TGAATCCTCGGCCTTCTATG
Gif-Rev	CAGTTAAAGTTGGTGGCACTT
Muc6-For	TGCATGCTCAATGGTATGGT
Muc6-Rev	TGTGGGCTCTGGAGAAGAGT
Tff2-For	ACCCGGGCATCAGTCCCGA
Tff2-Rev	GCAGCTCCCAGGGAACGGGT
Muc1-For	AGTACCAAGCGTAGCCCCTA
Muc1-Rev	AAGGGCATGAACAGCCTACC
Muc5AC-For	CATGACCTGTTATAGCTCCGA
Muc5AC-Rev	CTCAGTAACAACACAGCCTC
Cldn18-For	GCCTGTCTCTTGTCCTCTCC
Cldn18-Rev	CAGGTTGGCGGGTCTTAGAGC
Shh-For	TGATCCTTGCTTCCTCGCTG
Shh-Rev	CACCTCTGAGTCATCAGCCG
H+/K+ ATPase-For	GTTCCAGTGGTGGCTGGT
H+/K+ ATPase-Rev	GCTGATAGTGGATGGAGAGATG
Hprt-For	TCCTCAGACCGCTTTTTGCC
Hprt-Rev	GTGATGGCCTCCCATCTCCT

Primers for real time quantitative PCR

Primer	Sequence
Cdx2-For	TCCCTAGGAAGCCAAGTGAAA
Cdx2-Rev	AGTGAAACTCCTTCTCCAGCTC
Olfm4-For	GCCACTTTCCAATTTCAC
Olfm4-Rev	GAGCCTCTTCTCATACAC
Dach1-For	TTGAGACAAAACGCCGTGAG
Dach1-Rev	CGGTCAGCTTCTATCTCAGGG
Clca4-For	CCTGGAGGCTGAGTTTATAGGTG
Clca4-Rev	GAGCCAGAGAATGCCCACTC
Smoc2-For	ACAAGTCCATCACCGTGCAG
Smoc2-Rev	CAGCCGTTCATCCTTGTTTCC
Cdca7-For	GGAGTCCTGGTGTATCTGGC
Cdca7-Rev	ACGTCGAGAACAAGAGAGCC
Msi1-For	GGTTTCGGCCACAGTCTTG
Msi1-Rev	CTGGCTCAGTCTGGTCCTC
Gif-For	CCTGGGGCCTTATTGTCTCTTC
Gif-Rev	TGAAGTTGGCTGTGATGTGC
Col11a2-For	GATGAGCTGAGCCCTGAGAC
Col11a2-Rev	CTGCTCCAGTACAGGCGTG
Pgc-For	TGCCTACCCTCACTTTTGTCC
Pgc-Rev	CACTCTCAGCGTTCAGGGAG
Muc2-For	GAACGGGGCCATGGTCAGCA
Muc2-Rev	CATAATTGGTCTGCATGCC
Cdh17-For	CAAGTCTGTGCACCAAGCAC
Cdh17-Rev	TGCCATAGCCAAGTTGGAGG
Reg4-For	GCCTTGCACAAACTCTTCCC
Reg4-Rev	TGGTTTGACAGAGACGCAGT
Cdx1-For	GCTCACAGAGCGGCAGGTAA
Cdx1-Rev	GGTGTGGGAGTGCCATCCAG
Muc6-For	TGCATGCTCAATGGTATGGT
Muc6-Rev	TGTGGGCTCTGGAGAAGAGT
Tff2-For	ACCCGGGCATCAGTCCCGA
Tff2-Rev	GCAGCTCCCAGGGAACGGGT
Cldn18-For	GCCTGTCTCTTGTCCTCTCC
Cldn18-Rev	CAGGTTGGCGGGTCTTAGAGC
Gapdh-For	TTCACCACCATGGAGAAGGC
Gapdh-Rev	GGCATGGACTGTGGTCATGA
